# Mitochondrial dynamics and metabolic regulation control T cell fate in the thymus

**DOI:** 10.3389/fimmu.2023.1270268

**Published:** 2024-01-15

**Authors:** Rima Elhage, Mairead Kelly, Nicolas Goudin, Jérôme Megret, Agnès Legrand, Ivan Nemazanyy, Cécilia Patitucci, Véronique Quellec, Timothy Wai, Ahmed Hamaï, Sophie Ezine

**Affiliations:** ^1^ Institut Necker Enfant-Malades (INEM), INSERM U1151-CNRS UMR 8253, Université de Paris, Paris, France; ^2^ Platform for Image Analysis Center, SFR Necker, INSERM US 24 - CNRS UMS 3633, Paris, France; ^3^ Platform for Cytometry, SFR Necker, INSERM US 24 - CNRS UMS 3633, Paris, France; ^4^ Platform for Metabolic Analyses, SFR Necker, INSERM US 24 - CNRS UMS 3633, Paris, France; ^5^ Mitochondrial Biology Group, Institut Pasteur, CNRS UMR 3691, Paris, France

**Keywords:** thymus, T cell progenitors, β-selection checkpoint, mitochondrial dynamics, metabolome, glycolysis, OxPhos

## Abstract

Several studies demonstrated that mitochondrial dynamics and metabolic pathways control T cell fate in the periphery. However, little is known about their implication in thymocyte development. Our results showed that thymic progenitors (CD3^-^CD4^-^CD8^-^ triple negative, TN), in active division, have essentially a fused mitochondrial morphology and rely on high glycolysis and mitochondrial oxidative phosphorylation (OXPHOS). As TN cells differentiate to double positive (DP, CD4^+^CD8^+^) and single positive (SP, CD4^+^ and CD8^+^) stages, they became more quiescent, their mitochondria fragment and they downregulate glycolysis and OXPHOS. Accordingly, *in vitro* inhibition of the mitochondrial fission during progenitor differentiation on OP9-DL4 stroma, affected the TN to DP thymocyte transition by enhancing the percentage of TN and reducing that of DP, leading to a decrease in the total number of thymic cells including SP T cells. We demonstrated that the stage 3 triple negative pre-T (TN3) and the stage 4 triple negative pre-T (TN4) have different metabolic and functional behaviors. While their mitochondrial morphologies are both essentially fused, the LC-MS based analysis of their metabolome showed that they are distinct: TN3 rely more on OXPHOS whereas TN4 are more glycolytic. In line with this, TN4 display an increased Hexokinase II expression in comparison to TN3, associated with high proliferation and glycolysis. The *in vivo* inhibition of glycolysis using 2-deoxyglucose (2-DG) and the absence of IL-7 signaling, led to a decline in glucose metabolism and mitochondrial membrane potential. In addition, the glucose/IL-7R connection affects the TN3 to TN4 transition (also called β-selection transition), by enhancing the percentage of TN3, leading to a decrease in the total number of thymocytes. Thus, we identified additional components, essential during β-selection transition and playing a major role in thymic development.

## Introduction

Emerging studies highlight the involvement of cellular and mitochondrial metabolism and the role of mitochondrial dynamics in the function and fate of mature T cells (1–4). However, the implication of metabolism and mitochondrial dynamics in thymic development are still poorly evaluated.

The development of triple negative thymic progenitors (TN, CD3^-^CD4^-^CD8^-^; TN1 to TN4) is highly regulated and involves multiple proliferation and selection events prior to the emergence of naïve T cells from the thymus (5–8). This development implies rearrangements of the TCR genes and the β-selection program. TCR rearrangements begin at the TN2 stage (CD3^-^CD44^+^CD25^+^), continue and end at stage TN3. The β-selection occurs at the TN3 stage and is a process in which thymocytes successfully expressing a pre-TCR complex consisting of rearranged TCRβ and pre-TCRα, are rescued from cell death and allowed to proliferate significantly and differentiate into TN4 cells. TN3 cells that do not have the pre-TCR complex die by apoptosis. TN4 cells enter a state of intense proliferation and differentiate into double-positive CD4^+^CD8^+^ cells (DP), at which stage rearrangements of the locus encoding the TCRα chain and positive and negative selection occurs.

The important proliferation during β-selection serves to increase the diversity of the TCR repertoire and is signaled through the pre-TCR and stromal-derived factors: Delta-like 4 (DL4), non-redundant ligand for Notch1, and Interleukin-7 (IL-7) (9–13).

In response, TN3 switch from quiescence to active cycling and use pathways to increase nutrient uptake in order to fuel repeated proliferations. Several of these pathways have been well described. The PI3K/3-phosphoinositide-dependent protein kinase 1/Akt/mTORC signaling is likely to be particularly important for cellular survival, proliferation and differentiation at the β-selection checkpoint (14–16). Moreover, the serine/threonine kinase (LKB1) has an essential role in T cell progenitor proliferation (15, 17). Both of these pathways promote T cell growth by acting on the expression of nutrient receptors: the glucose transporter Glut1, CD71 (transferrin receptor, key receptor for the uptake of iron), CD98 (component of the L-amino acid transporter), which are highly expressed in T cell progenitors TN3 and even more in TN4, demonstrating a critical role for glucose and amino acid (AA) uptake and glycolytic metabolism in these T cell subsets (9, 12, 16, 18).

This allows the TN3 and TN4 subsets to acquire enough nutrients such as glucose or glutamine to support cellular metabolism, energy and biosynthetic needs such as *de novo* DNA, protein (AA) and lipid (fatty acid) synthesis; all of which are crucial for early T lymphocyte development in the thymus (19).

Mitochondrial dynamics are closely linked to cell metabolism. Although changes in the ultrastructure of the mitochondria (fusion/fission) have long been observed in response to alterations in oxidative metabolism (20), it has become increasingly clear that changes in mitochondrial morphology can also dramatically effect cellular and organismal metabolism (3, 4, 21–23).

Mitochondria are highly dynamic organelles that move continuously, divide and fuse in a highly regulated manner. The balance between these opposite processes of mitochondrial fusion and fragmentation are executed by mitochondrial shaping dynamin-like GTPases. Besides their implication in metabolism, evidence is accumulating on their role in several other functions: cell proliferation (24–26), death (21), migration (27), production of reactive oxygen species (ROS) (28) and mitophagy (29). Interestingly, all these processes are essential for correct function of the adaptive immune system and thymic development.

Intrathymic development is also strongly associated with the expression of mitochondrial shaping proteins (4, 23). The deletion of mitochondrial pro-fission protein Drp1 reduced the number of developing thymocytes and mature T cell homeostasis by regulating their number without affecting differentiation (4). Moreover, deletion of mitochondrial pro-fusion protein Opa1 during early thymocyte maturation reduced mitochondrial metabolism, impaired thymocyte development at the β-selection stage and induced a threefold reduction of the total number of thymocytes (23).

Despite all these observations, mitochondrial morphology in the different thymic T cell progenitors and mature T cells and how this morphology is associated with metabolic changes and regulates T cell development remains unclear. The aim of our work is to characterize the cellular and mitochondrial metabolism as well as mitochondrial dynamics in the different thymic T cell populations, and to evaluate if changes in cellular and/or mitochondrial metabolism or morphology could have a direct effect on proliferation and/or differentiation of thymic lymphocytes. Here, we have characterized the main metabolic steps, as well as mitochondrial dynamics during T cell development and their association regarding thymic cellularity.

## Materials and methods

### Mice and *in vivo* treatment

C57BL/6J Ly5.1, IL7Rα knockout mice were purchased from CDTA (Orleans, France). ROSA26lox-Stop-lox-mito-YFP (referred to as Mito-YFP hereafter), a reporter mouse with ubiquitous expression of YFP targeted to the mitochondrial matrix (30), with a C57BL/6N background and their control C57BL/6N were a gift from Nils-Göran Larsson from the Max Planck Institute and Karolinska Institute. Mice were kept in the specific pathogen-free facility of SFR Necker (Agreement n°75-1026) and used at 6–8 weeks of age (males and females). All experimental procedures using animals were approved by Paris Descartes University Ethical Committee and the French Ministry of Research, Innovation and Education under the following reference APAFIS #26599-2019071812345604.

To follow the involvement of glycolysis in differentiation and/or thymic proliferation, we inhibited glycolysis *in vivo*. Two groups of 4 mice were treated, by intraperitoneal injections of either phosphate buffered saline (PBS) or 2-DG (2-deoxyglucose, Sigma, Cat. 154-17-6) at the dose of 750 mg/kg every 2 days for 2 weeks. 2-DG is a glucose analogue that acts as a competitive inhibitor of glucose metabolism via its actions on hexokinase, the limiting enzyme of the glycolysis pathway. It is phosphorylated in 2-DG-P which cannot be metabolized by the phospho-glucose isomerase. This leads to the accumulation of 2-DG-P in the cell and the depletion of cellular ATP.

### Staining and sorting of murine thymocytes

Thymi were dissociated through a 45-μm cell strainer. Thymocytes from Mito-YFP mice were processed into a mononuclear cell suspension, and sorted by flow cytometry into the 5 thymic cell subsets (TN3, TN4, DP CD4^+^CD8^+^, and SP CD4^+^TCRβ^+^ and CD8^+^TCRβ^+^ cells). Thymocytes were first stained with Live/Dead cell viability dyes (1/1000) (ThermoFisher Scientific) for 10 min in PBS at room temperature, cells were then washed and stained for 30 minutes with the following antibodies (Abs): anti-CD4 (RM4-5), anti-CD8β (H35-172), anti-CD44 (IM7), CD25 (IL-2Rα chain, PC61) and lineage mixture (containing the following Abs: anti-CD11b/Mac-1 (M1/70), anti-CD11c (N418), anti-Ly6G/Ly6C (RB6-8C5), anti-CD19 (1D3), anti-NK1.1 (PK136), anti-TCRβ (H57-597), anti-TCRγδ and anti-Ter119/Erythroid cells (TER119) obtained from BD Biosciences and Sony Biotechnologies. The cells were then analyzed and/or sorted (**Supplementary Figure 1**). Flow cytometry data were acquired using FACS Canto II running the DIVA software (BD Biosciences). Cell sorting was performed using FACS Aria III running the DIVA software (BD Biosciences). Flow cytometry data analysis was performed using the FlowJo software (BD Biosciences).

For thymocyte proliferation analysis, cells were stained with anti-Ki67 (16A8). The analysis of mitochondrial membrane potential was carried out using TMRE (tetramethylrhodamine, ethyl ester), a fluorescent cationic cell-permeable dye. The uptake of TMRE into mitochondria depends on mitochondrial membrane potential and can therefore be used as a surrogate to estimate mitochondrial activity. The analysis of mitochondrial mass was carried out using Mito-Tracker Green, a fluorescent cell-permeable dye, trapped by mitochondria in live cells, independently of mitochondrial membrane potential. The analysis of glucose uptake in live cells, was carried out using 2-NBDG, 2-deoxy-2-[(7-nitro-2,1,3-benzoxadiazol-4-yl) amino]-D-glucose, a fluorescent tracer, derivative of glucose.

For Seahorse and Western Blot analysis, approximately 12 to 15 Mito-YFP mice were used and 1 to 1.5x10^7^ thymocytes were obtained and separated into 3 fractions: the first fraction was enriched with thymic CD4 and progenitor cells, by untouched selection, using anti-CD8α (Lyt2), anti-Ter119 (Ter119) and anti-Ly6G/Ly6C (RB6-8C5) to remove the unwanted DP, erythroid and granulocytes cells. The second fraction was enriched with thymic CD8 and progenitor cells, by untouched selection using anti-CD4 (GK1.5), anti-Ter119 and anti-Ly6G/Ly6C. The third fraction was used to directly sort DP cells. The CD4 or CD8 enriched fractions were then stained and sorted to obtain TN3, TN4 and CD4 or CD8 cells respectively.

### Thymocyte OP9-DL4 coculture

For *in vitro* assays, 20,000 sorted TN3 cells (Lin^-^CD44^-^CD25^+^) were washed and resuspended in Opti-MEM medium containing 10% Fetal Bovine Serum (FBS), 1% penicillin (100U/ml) and streptomycin (100μg/ml). The cells were co-cultured in a 24-well plate on 80%-confluent monolayers of OP9-Delta-like-4 (DL4) cell lines in the presence of 1 ng/ml IL-7 and 5 ng/ml of the corresponding Flt3 ligand (16, 31). OP9-DL4 cells are generated from the bone marrow stromal cell line OP9 and allow *in vitro* T-cell commitment and differentiation through Notch receptor signaling (32, 33).

Cells were co-cultured during 5 and 10 days, in the presence or absence of chemical molecules Mdivi-1 and M1. Mdivi-1 (for mitochondrial division inhibitor) is a quinazonilone derivative that was reported to inhibit Drp1 (dynamin-related protein)-dependent mitochondrial fission (34) and M1 (Mitochondrial Fusion Promoter) is a cell permeable hydrazone that enhances mitochondrial fusion (35). Two doses of Mdivi-1 at 2.5 and 5μM and two doses of M1 at 10 and 20μM were used at day 0 and every 3 days.

### Seahorse or extracellular flux analysis

To assess the metabolic flux dynamics in thymic cell subsets, we used the Seahorse XFe96 bioenergetic analyzer (Agilent Technologies, USA) that simultaneously, and in real time, measures the two main energy pathways of the cell (glycolysis and mitochondrial respiration) on live cells in a non-invasive manner. It measures the oxygen consumption rate (OCR), an indicator of mitochondrial oxidative phosphorylation (OXPHOS) - as well as extracellular acidification rate (ECAR) an indicator of glycolytic metabolism. Briefly, 1 hour before the test, the 5 thymic flow cytometry-sorted cell subsets from Mito-YFP mice: TN3, TN4, DP CD4^+^CD8^+^ cells, and SP CD4^+^TCRβ^+^ and CD8^+^TCRβ^+^ cells were washed in unbuffered XF assay media (Agilent technologies) supplemented with either 2 mM glutamine, 25 mM glucose and 1 mM sodium pyruvate for OCR analysis or 2 mM glutamine for ECAR analysis, and were seeded at a density of 0.4 or 0.8 million cells per well for OCR and ECAR, respectively, in specialized 96-well plates (Seahorse XF96 V3 PS microplate, 101085-004, Agilent technologies). The cells were then centrifuged and incubated for 1 hour at 37°C in a CO_2_-free incubator. OCR was measured under basal conditions and in response to 1 μM oligomycin (inhibitor of ATP synthase, the complex V of the electron transport chain (ETC)), 1 μM carbonyl cyanide p-trifluoromethoxyphenylhydrazone (FCCP) which uncouples ATP synthesis from ETC and stimulates the respiratory chain to operate at maximum capacity and 1 μM rotenone + 1 μM antimycin A (inhibitors of ETC complex I & III respectively). ECAR was measured under basal conditions and in response to 10 μM glucose, 1 μM oligomycin and 50 mM 2-deoxyglucose (2-DG, glycolysis inhibitor).

### Western blotting

For western blot analysis, sorted cell subsets were washed twice with ice cold PBS and lysed in an appropriate lysis buffer (50 mM Tris/HCl, pH 7.5, 100 mM NaCl, 1% Triton X-100, 0,5% NP-40, 1% protease and phosphatase inhibitor cocktail (78442, Thermo Fisher Scientific)). Protein extracts were denatured and separated by SDS-PAGE. Proteins were transferred onto a PVDF membrane and probed with primary antibodies, followed by the appropriate horseradish peroxidase HRP-conjugated secondary antibody. Signals were quantified using Image J software and normalized to GAPDH for each sample. The following primary antibodies were used: HK1/Hexokinase-1 (sc-46695, Santa Cruz Biotechnology, 1:1000), HK2/Hexokinase-2 (2867, Cell Signaling, 1:1000), PKM2/Muscle Pyruvate Kinase 2 (4053, Cell Signaling, 1:1000), TOMM40/Translocase of outer mitochondrial membrane 40 (18409-1-AP, Proteintech Europe, 1:1000) and GAPDH (sc-25778, Santa Cruz Biotechnology, 1:1000).

### Targeted metabolomic analysis by liquid chromatography mass spectrometry

We measured numerous polar metabolites using liquid chromatography coupled with mass spectrometry (Q Exactive Plus Orbitrap mass spectrometry, ThermoFischer scientific) capable of measuring mass-to-charge ratios, enabling the identification and quantitation of approximately 140 metabolites, including AA, intermediates of the glycolytic cycle and Krebs cycle.

The 5 thymic cell subsets were washed with ice cold PBS, flash-frozen and stored at -80°C until extraction with an adequate volume (500.000 cells/ml) of an aqueous solution of methanol and acetonitrile.

The analysis of metabolome data was carried out by a free Canadian platform ‘MetaboAnalyst’ made available to the scientific community (www.metaboanalyst.ca).

### Confocal microscopy analysis

To avoid apoptosis during cell sorting, the thymocytes were marked by a cocktail of antibodies, quickly fixed by paraformaldehyde, sorted and placed on poly-lysine coated slides. This attachment step allowed to preserve mitochondrial morphology, which is known to be hypersensitive to external variations. Cells were acquired as Z-stack images by confocal microscopy (Zeiss LSM700) at objective 63x oil NA 1.4. allowing a 3D reconstruction of the mitochondria and good visualization of their fragmented or fused shape. Although methods for correct imaging and quantification of mitochondrial morphology have improved with the generalization of computational tools that allow more automatic and quantitative approaches (36), they were not reliable in our study. Indeed, we were hindered by the size of the cells as lymphocytes measure less than 10 µm with a large nucleus and very restricted cytoplasm, preventing the obtention of sufficiently precise results with an automatic approach. Consequently, we scored the number of cells with fragmented, fused or both fragmented and fused mitochondria as described by Lebeau et al. (37). We consider mitochondria as fragmented when their shape is small and round (**Supplementary Figure 2** and **Supplementary Movie 1**). Fused mitochondria are characterized by elongated shape and networtk (**Supplementary Figure 2** and **Supplementary Movie 1**). This scoring was performed using ICY software v2.4.3 (38) on each individual 3D cell. At least 50 cells per lymphocyte population were analyzed in 3 independent experiments of measurement of mitochondrial shape.

### Quantification and statistical analysis

Prism Software was used for analysis. Data are expressed as mean ± SEM. One way and two-way ANOVA was used for multiple comparison (with *post hoc* Holm-Sidak’s test). P values are indicated in the figures as follows: * = p < 0.05, ** = p < 0.01, *** = p < 0.001, **** = p < 0.0001. Comparison between two groups was performed using homoscedastic unpaired two-tailed student’s t test. For all experiments, n (number of mice used) is indicated in the figure legends.

## Results

### High oxidative metabolism and glycolysis in thymic T cell progenitors

Changes in mitochondrial metabolism have been shown to be involved in mature T cell differentiation (3). In order to extend these findings, we explored the mitochondrial metabolism during thymocyte development. We first evaluated: 1) the mitochondrial membrane potential (ΔΨm) (TMRE probe) in the different thymic populations, which reflects mitochondrial activity and ability to generate ATP by OXPHOS, and 2) the mitochondrial mass (Mitotracker Green probe). We quantified these probes by measuring their median fluorescence intensity (MFI). For most of our comparisons, the TN4 subset (the last stage of progenitor’s development) served as reference to which the other populations were compared.

**Figure 1:**
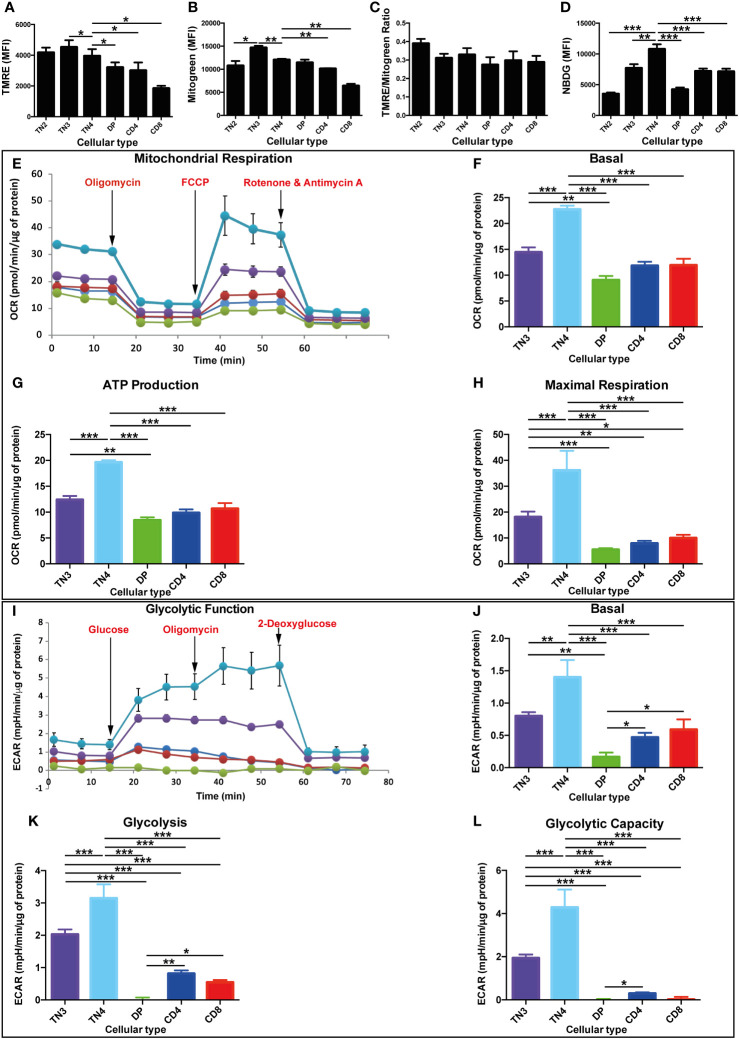
High oxidative and glycolysis metabolism in T progenitors in comparison with mature T thymic cells. Mitochondrial mass, membrane potential, glucose absorption and energy pathways in different thymic lymphocyte populations in Wild Type (WT) mice. **(A–L)** correspond to data from a representative experiment (n=3 independent experiments). Each experiment represent average ± SEM of 4 to 5 mice. The mean of each population is compared to TN4 population. **(A)** Quantification of mitochondrial mass by Mitotracker Green median fluorescence intensity (MFI). **(B)** Quantification of mitochondrial membrane potential by TMRE MFI. **(C)** Analysis of the membrane potential/mitochondrial mass ratio. This ratio takes into account differences in mitochondrial mass between different cell populations. **(D)** Quantification of glucose absorption by 2-NBDG MFI. **(E–L)** correspond to data from a representative experiment (n=3 independent experiments). Each experiment represents average ± SEM of 6 individual wells of sorted cells from 13 to 15 mice. **(E)** OCR of different thymocyte populations under basal conditions (initial rates) and in response to sequential treatments with oligomycin, FCCP and rotenone/antimycin **(A)** The arrows indicate the time (min) of addition of each reagent. **(F)** basal OCR and **(G)** production of ATP, representing the oxidative phosphorylation of different populations of thymocytes expressed as a histogram. **(H)** maximum respiration of different populations of thymocytes expressed as a histogram. **(I)** ECAR of different thymocyte populations under basal conditions (initial levels) and in response to sequential treatments with glucose, oligomycin and 2-deoxyglucose. **(J)** basal ECAR **(K)** glycolysis, after treatment with glucose and **(L)** glycolytic capacity, of the different populations of thymocytes expressed as a histogram. OCR and ECAR levels are expressed per µg of protein. P values are indicated in the figures as follows: * = p < 0.05, ** = p < 0.01, *** = p < 0.001.

Our results show that, the MFI of both dyes increased during TN2 to TN3 transition (not statistically significant for TMRE and p<0.05 for Mitotracker Green), reached a maximum at the TN3 stage and significantly decreased in TN4 (p<0.05 for TMRE and p<0.01 for Mitotracker Green). Thus, ΔΨm and mitochondrial mass decreased during β-selection (**Figures 1A, B**).

As the TN thymocyte progenitors mature to DP and SP CD4^+^TCRβ^+^or CD8^+^TCRβ^+^ cells, we also observed a decrease in mitochondrial mass and ΔΨm, thus reflecting a decrease in mitochondrial function (**Figures 1A, B**). In mature SP thymocytes, Mitotracker Green and TMRE staining were both higher in CD4^+^TCRβ^+^ than in CD8^+^TCRβ^+^. We calculated the membrane potential/mitochondrial mass ratio, and observed no variation, suggesting a positive correlation between mitochondrial activity and mitochondrial mass in all thymic subsets (**Figure 1C**).

Therefore, TN3 and TN4 thymic pre-T cells have a higher mitochondrial mass and seem to have more active/functional mitochondria in comparison to mature thymocytes. This is in relation to their progenitor status undergoing metabolic reprogramming to provide energy and important molecular building blocks for growth, proliferation, and differentiation.

Another important characterization of cell population metabolism is glucose uptake. Hence, we used 2-NBDG, 2-deoxy-2-[(7-nitro-2,1,3-benzoxadiazol-4-yl) amino]-D-glucose, a fluorescent tracer used to monitor glucose uptake in live cells. The MFI quantification of NBDG showed an increase during the TN2 to TN4 transition and reached a maximum at the TN4 stage. It decreased in DP and SP CD4^+^TCRβ^+^ and CD8^+^TCRβ^+^ cells revealing the lowest level at the DP stage (**Figure 1D**). Thus, glucose uptake is essential for thymic pre-T cells and specifically at the TN4 stage, which led us to analyze dynamic fluxes.

To assess the metabolic flux dynamics during thymic development, we measured cells’ oxygen consumption rate (OCR) and extracellular acidification rate (ECAR), indicators of mitochondrial OXPHOS and glycolysis respectively, by using a Seahorse Flux Analyzer. The comparison of the 5 purified thymic cell subsets revealed that the TN4 progenitors displayed a strong basal OCR (25 pmol/min/μg of protein) compared to TN3 (15 pmol/min/μg protein) and mature T cells: DP, SP CD4^+^TCRβ^+^ and SP CD4^+^TCRβ^+^ (around 10 pmol/min/μg protein) (**Figures 1E, F**). TN3 cells presented a significantly higher basal OCR compared to DP. Oligomycin inhibits mitochondrial complex V (ATP synthase) of the OXPHOS system, and injection of this compound shows how much of the OCR is due to complex V activity. The values of ATP production in the different thymic populations follow the same trend as the basal OCR (**Figures 1E, G**). Moreover, TN3 and TN4 cells displayed a greater maximal respiratory capacity, respectively 4 and 7 times higher than in mature cells (**Figures 1E, H**). The basal ECAR was significantly higher in TN3 and TN4 cells (between 1 and 1.5 mpH/min/μg protein, respectively), compared to DP cells (0.2 mpH/min/μg protein) and CD4^+^TCRβ^+^ and SP CD4^+^TCRβ^+^ cells (between 0.5 and 0.6 mpH/min/μg protein, respectively) (**Figures 1I, J**). DP cells’ basal OCR, ATP production and maximal respiration were almost identical to those observed in SP cells (**Figures 1E–H**). However, their basal ECAR, aerobic glycolysis and glycolytic capacity decreased drastically compared to SP cells, and was barely quantifiable (**Figures 1I–L**). Interestingly, TN4 cells had a significantly higher basal ECAR than TN3 cells. After injection of glucose, the values of aerobic glycolysis followed the same trend as the basal ECAR, showing higher glycolysis in TN4 compared to TN3 cells and extremely low values in mature cells (**Figures 1I, K**). Similar to OCR values, TN3 and TN4 pre-T cells demonstrated the highest glycolytic capacity compared to mature thymocytes (**Figures 1I, L**).

### Fused mitochondria characterize thymic TN progenitors while mature thymocytes present fragmented mitochondria

**Figure 2:**
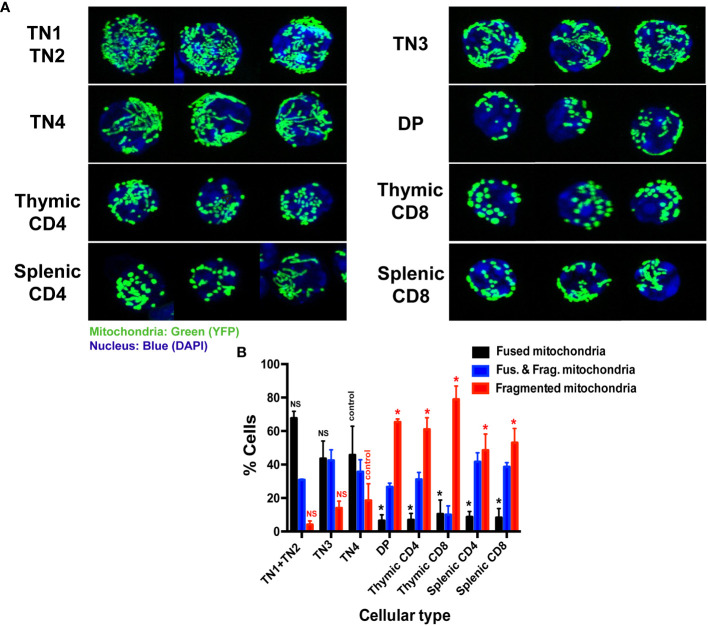
Thymic triple negative progenitors have fused mitochondria while mature T cells have fragmented mitochondria. **(A)** Representative confocal images of mitochondrial YFP fluorescence in freshly isolated thymocytes from Mito-YFP mice, showing mitochondrial morphology in the different thymic lymphocyte populations (TN1 and TN2, TN3, TN4, DP and SP CD4 or CD8 and splenic CD4 and CD8) isolated by cell sorting. Mitochondria are shown in green (YFP) and nuclei in blue (DAPI). **(B)** Analysis of the percentage of cells expressing exclusively fused, exclusively fragmented mitochondria or both fused and fragmented mitochondria. Data represent mean ± SEM from 3 independent experiments (n=3). P values are indicated in the figures as follows: * = p < 0.05. NS: not significant.

The higher mitochondrial activity and glycolysis in pre-T cells, in comparison to mature thymocytes, led us to study their mitochondrial morphology (**Figure 2A**). Among each thymocyte population, we quantified the percentage of cells that had either fused, or fragmented mitochondria or both. At the progenitor stage, mitochondria were essentially fused (**Figure 2A**). Among a pool of sorted TN1 and TN2 cells, 66% presented fused mitochondria, 30% both fused and fragmented mitochondria and only 4% fragmented mitochondria (**Figures 2A, B**). Among the activated TN3 and TN4 cells that underwent metabolic reprogramming: 44 to 46% had fused mitochondria, 36 to 42% both morphologies and about 14 to 18% fragmented mitochondria (**Figures 2A, B**). As the cells differentiated into the DP and SP stages, the mitochondria became more fragmented (**Figures 2A, B**). The DP CD4^+^CD8^+^ had mostly small, fragmented, and dispersed mitochondria throughout the cell: 66% had fragmented mitochondria, 27% presented both morphologies and only 7% had fused mitochondria. Like the DP cells, mature thymocytes presented mainly fragmented mitochondrial. Among CD4^+^ cells: 62% had fragmented mitochondria, 31% both morphologies and only 7% of cells presented the fused morphology. The majority (80%) of SP CD8^+^ cells were fragmented, 10% had both fused and fragmented mitochondria and 10% a fused morphology. The peripheral splenocytes, CD4^+^ and CD8^+^ cells, presented a fragmented morphology, similar to SP populations in the thymus.

Altogether, these data indicate that intrathymic differentiation is represented by the transition of a fused mitochondrial morphology present in TN progenitors, to a fragmented one that takes place at the DP stage, and continues through SP and peripheral splenocytes where the majority of cells present fragmented mitochondria. Thus, mitochondrial morphology seems to be closely linked to T cell metabolism.

### Mitochondrial dynamics control the fate of T cells in the thymus

**Figure 3:**
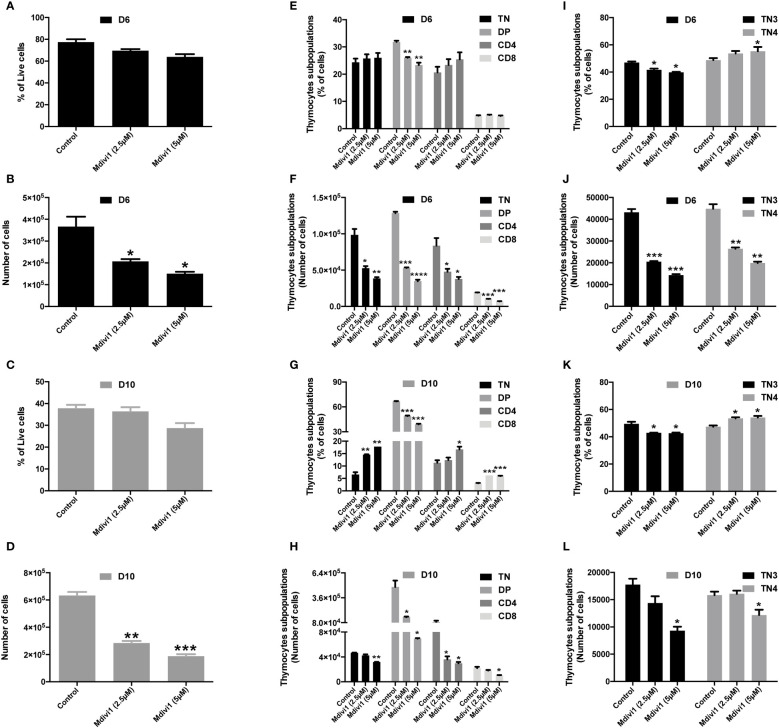
Modulation of mitochondrial dynamics of TN3 cells cultured in the presence of Mdivi-1 (an inhibitor of Drp1) affected the TN-DP transition and decreased the total number of differentiated thymocytes. Sorted TN3 cells co-cultured *in vitro* on OP9-DL4 feeder cells, in the presence of Mdivi-1 (2.5 μM and 5 μM). Mdivi-1 was added at day 0 then every 3 days for 10 days and analyzed on day 6 (D6) and day 10 (D10). Cells were marked with a cocktail of antibodies (anti-CD4, anti-CD8, anti-TCRβ, anti-LIN, anti-CD44 and anti-CD25) to assess the percentage of living cells at D6 **(A)** and D10 **(C)**; percentage of TN, DP, SP CD4^+^ TCRβ^+^ and SP CD8+ TCRβ^+^ cells at D6 **(E)** and D10 **(G)** TN3 and TN4 at D6 **(I)** and D10 **(K)**. Absolute number of living cells at D6 **(B)** and D10 **(D)**; TN, DP, SP CD4^+^ TCRβ^+^ and SP CD8^+^ TCRβ^+^ at D6 **(F)** and D10 **(H)**; TN3 and TN4 at D6 **(J)** and D10 **(L)**. Data from a representative experiment (n=3 independent experiments). Each experiment represents average ± SEM of 3 individual wells. Statistical significance is indicated as follows: *p < 0.05, **p < 0.01, ***p < 0.001, ****p < 0.0001.

Analysis of the mitochondrial morphology allowed to establish a mitochondrial mapping of each thymic population. During their differentiation, progenitors first displayed fused mitochondria and more mature populations fragmented ones. In order to evaluate whether the mitochondrial morphology impacted thymic proliferation and/or differentiation, our strategy was to inhibit mitochondrial fission or promote mitochondrial fusion during TN differentiation. Among the molecules used to modify fusion or fission, we used (i) a Drp1 inhibitor, Mdivi-1(Mitochondrial division inhibitor 1) and (ii) the mitochondrial fusion promoter M1. Thymic development could easily be manipulated by using an *in vitro* co-culture model of progenitor differentiation on OP9-DL4 feeder cells, this well characterized cell line allows commitment and differentiation of T cells through its ability to induce Notch signaling (32, 33). In the presence of mitochondrial fusion/fission modulators Mdivi-1 and M1, we tracked the ability of purified TN3 to perform β-selection, to generate TN4, and to differentiate into mature DP and SP thymocytes. The drugs were added at day 0 and every 3 days throughout cell culture. Our results showed that Mdivi-1 treatment did not affect the proportion of viable cells at day 6 (D6, **Figure 3A**) or day 10 (D10, **Figure 3C**), suggesting that Mdivi-1 did not impair cell viability compared to untreated cells. However, the total number of cells decreased markedly indicating an effect on proliferation and/or differentiation (**Figures 3B, D**).

The proportion of DP cells was reduced with Mdivi-1 treatment at D6 and D10, whereas the percentage of TN, CD4 and CD8 cells tended to increase at D6 (**Figure 3E**) and increased significantly at D10 (**Figure 3G**). The absolute number of TN, DP, CD4 and CD8 cells were, however, all reduced from D6 (**Figures 3F, H**). In line with this, TN3 cells treated with Mdivi-1 revealed that the proportion of TN3 cells was reduced at the 2.5 μM dose (p<0.05) and the 5 μM dose (p<0.05), whereas, the frequency of TN4 was slightly increased at the 2.5 μM dose (not statistically significant) and the 5 μM dose (p<0.05) at D6 (**Figure 3I**). We observe the same trend at D10, where the proportion of TN3 cells was reduced at both 2.5 μM and 5 μM doses (p<0.05), and the frequency of TN4 was slightly but significantly increased at both doses (p<0.05) (**Figure 3K**), demonstrating an acceleration, during differentiation, of TN3 to TN4. However, the absolute numbers revealed that both progenitor types decreased in culture at D6 (**Figure 3J**) and D10 (**Figure 3I**). Similar results were obtained using the mitochondrial fusion promoter M1 (**Supplementary Figure 3**). Thus, administration of pro-fusion molecules during T cell development affected the TN-DP transition. Therefore, the modification of mitochondrial morphology affected the development of thymic T cells during TN-DP transition, leading to a decrease in the total number of cultured cells. Our results recall data showing that in Drp1-deficient mice, the total number of thymocytes decreased and was associated with a decrease in thymic parenchyma, without any change in the percentage of each lymphocytic population of the thymus (TN1, TN2, TN3, TN4, DP and SP CD4 and CD8) (4).

### Thymic development is characterized by metabolomic rewiring. TN3 and TN4 are metabolomically distinct populations

**Figure 4:**
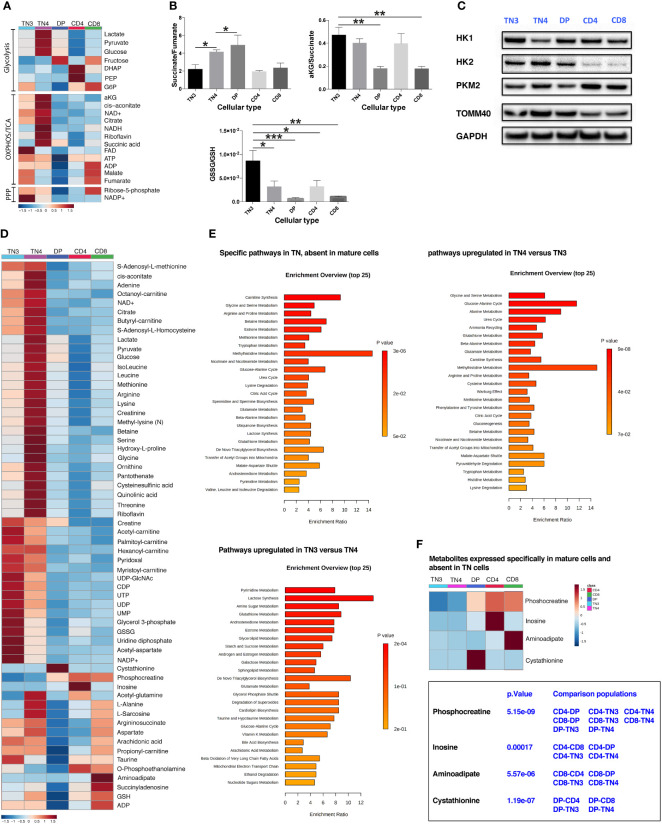
Thymic development is characterized by metabolomic rewiring. TN3 and TN4 are metabolomically distinct populations. **(A)** Heat map of the different metabolites related to glycolysis, OXPHOS, TCA cycle and the phosphate pentoses (PPP) pathway. **(B)** Histogram of the different ratios: succinate/fumarate, α-ketoglutarate (αKG)/succinate and GSSG/GSH to indicate whether cellular metabolism is primarily oxidative or glycolytic. Statistical analysis: one-way Anova, multiple comparisons, the mean of each population is compared to TN3 population. **(C)** Western Blot Analysis of 3 enzymes involved in the glycolysis pathway: hexokinase 1 and 2 (HK1, HK2) and muscle pyruvate kinase 2 (PKM2) in the different thymic lymphocyte populations (TN3, TN4, DP, CD4 and CD8). TOMM40 (translocase of outer mitochondrial membrane 40) is an outer mitochondrial membrane protein revealing the mitochondrial mass and the housekeeping protein GAPDH (glyceraldehyde 3-phosphate dehydrogenase) is used as loading control. **(D)** General heat map of the targeted metabolite set. **(E)** Differentially expressed pathways, that specifically characterize: TN cells (absent in mature cells) (top left panel); TN4 cells (up-regulated compared to TN3 cells) (top right panel) and TN3 cells (up-regulated compared to TN4 cells) (bottom left panel). **(F)** Heat map of metabolites specific for mature cells and absent in TN cells. Data represent mean ± SEM (n=5 to 6 samples for each cell population) from 2 independent experiments. P values are indicated in the figures as follows: * = p < 0.05, ** = p < 0.01, *** = p < 0.001.

In order to define a metabolic signature and to understand how different metabolic processes influence the differentiation and/or the proliferation of thymic subsets, we measured numerous polar metabolites (approximately 140 metabolites), including AA, intermediates of the glycolytic cycle and tricarboxylic acid cycle (TCA cycle), using LC-MS. Among, these 140 metabolites, 115 were retained for analysis (**Supplementary Table 1**). This targeted metabolomic approach identified 87 molecules that were significantly modulated between the different thymocyte populations (**Supplementary Table 1**). For our evaluation, we first focused on the OXPHOS/TCA cycle and glycolysis-related metabolites. As shown in **Figure 4A**, illustrating the ‘heat map’ of metabolite levels in cells, TN4 cells revealed a higher level of glucose, pyruvate and lactate correlating with a higher glycolytic flow compared to other cells, including TN3. We also observed a higher level of TCA intermediates, with the exception of fumarate and malate in TN4 cells. While the α-ketoglutarate (α-KG)/succinate ratio was unchanged, TN4 cells displayed a higher succinate/fumarate ratio compared to TN3 cells (**Figure 4B**). In contrast, TN3 cells exhibited higher levels of fumarate and malate suggesting a greater activity of succinate dehydrogenase (SDH, mitochondrial complex II) coupled with the mitochondrial respiratory chain or OXPHOS. This was reinforced by an increase in the level of FAD and ATP in TN3 cells compared to TN4 cells (**Figure 4A**). In addition, TN3 cells exhibited a higher oxidative state compared to others cellular contingents (TN4, DP, CD4 and CD8) as indicated by two major redox couples. Indeed, TN3 cells demonstrated higher levels of NADP and an increased GSSG (oxidized glutathione)/GSH (reduced glutathione) ratio (**Figures 4A, B**). In summary, the analysis of the metabolome showed that TN4 were more glycolytic than TN3, and that TN3 relied more on oxidative phosphorylation. It is noteworthy that DP displayed an accumulation of succinate (confirmed by the ratios of succinate/fumarate and α-KG/succinate in **Figure 4B**) in contrast to CD4 and CD8 cells.

Glucose metabolism is a multi-step process, controlled by several enzymes. Among them, the hexokinase (HKs) enzymes (HK1 and HK2) and phosphofructokinase 1 (PFK-1) control 2 key stages of glycolysis. HKs catalyze the first step of glucose metabolism by converting glucose into glucose-6-phosphate. HK can be expressed as four different isoforms, the main ones are HK1 and HK2; HK1 being a somewhat ubiquitous isoform and HK2 existing as a more selectively regulated isoform (39). HK2 is also located at the mitochondrial outer membrane and can have direct access to ATP. Western Blot analysis in TN3, TN4, DP, CD4 and CD8 thymic cells showed that they all expressed both forms of HK (**Figure 4C**). HK1 was ubiquitously expressed in all our thymic cell populations, but was very weak in TN4 cells compared to other cells. Inversely, HK2, which displays two active sites and is therefore more active than HK1, was strongly expressed in TN4 cells supporting the fact that TN4 cells are more glycolytic than TN3 (**Figure 4C**). Interestingly, in primary lymphocytes, it has been shown (40) that a rapid decline in glucose metabolism correlates with a decrease in HK2 expression. Inhibition of HK2 activity by chemical inhibitors or RNA interference decreases glucose use as well as ATP levels; however, its overexpression restores glucose retention. In addition, the expression of PKM2, an enzyme that occurs in the last stage of glycolysis and converts phosphoenolpyruvate into pyruvate was overexpressed in TN4 cells compared to TN3 cells. The expression of TOMM40 (translocase of outer mitochondrial membrane homolog 40) protein supports that the mitochondrial mass decreased during thymocyte development (as previously described in **Figure 1A** with Mitotracker Green staining).

The general heat map of all identified metabolites (**Figure 4D**) showed that there is a metabolic switch between different cell types; for the majority of these metabolites, their values were higher in TN4 progenitors compared to other cells, including TN3 progenitor cells, also supporting that TN4 are metabolically more active. If we consider the two groups together (TN3 and TN4), among the top-enriched pathways we observed: - mitochondria-associated pathways including L-carnitine biosynthesis involving transport of long chain fatty acids into the mitochondria via the carnitine shuttle and favoring fatty acid β-oxidation, the TCA cycle, transfer of acetyl groups into mitochondria, the malate-aspartate shuttle; - urea cycle straddling mitochondria and cytosol; - amino acid metabolisms (particularly including glycine, serine, arginine, proline, betaine, methionine, tryptophan, glutamate, branched chain AAs) that also fuel the TCA cycle; - antioxidant pathways including glutathione metabolism and ubiquinone biosynthesis; and - pyrimidine metabolism indispensable for nucleic acids, phospholipid, and glucose metabolism (**Figures 4D, E**).

Among the enrichment pathways which characterized TN4 cells compared to TN3 cells, we observed: the glucose-alanine cycle, amino acid metabolism (glycine, serine, alanine, glutamate, arginine, proline, cysteine, methionine), carnitine synthesis, the Warburg effect. However, the enrichment pathways which characterized TN3 cells compared to TN4 cells, were pyrimidine and glutathione metabolism (**Figure 4E**). Interestingly, phosphocreatine increased with thymocyte differentiation and characterized the DP, CD4 and CD8 cells and could have a critical role in generating the ATP pool and responding to the energy demand in these cellular populations (**Figure 4F**). This is in accordance with studies illustrating that creatine kinase B, which catalyzes the reversible transfer of the N-phosphoryl group from phosphocreatine to ADP to generate ATP, is significantly up-regulated during the differentiation of DP thymocytes into SP thymocytes (41, 42). CD4 and CD8 SP thymocytes were characterized by the expression of inosine (which is a nucleoside involved in the synthesis of adenine and in energy production) and aminoadipate (involved in lysine metabolism), respectively. DP cells, where there is a global shutdown of all metabolites, were characterized by cystathionine (an intermediary metabolite in cysteine synthesis from methionine in the transsulfuration pathway). Interestingly, cystathionine which is mainly considered to be a reservoir of cysteine for glutathione synthesis, may play an important role not only in thymopoiesis (43), but also in the protection against apoptotic cell death by mitigating endoplasmic reticulum stress (44), particularly in DP cells which are targeted by negative and positive selections in the thymus. Overall, we conclude that during intrathymic development, thymocyte cells are submitted to metabolomic rewiring and that TN3 progenitors are metabolomically distinct from TN4 cells.

### *In vivo* inhibition of glycolysis reduces thymic cellularity

**Figure 5:**
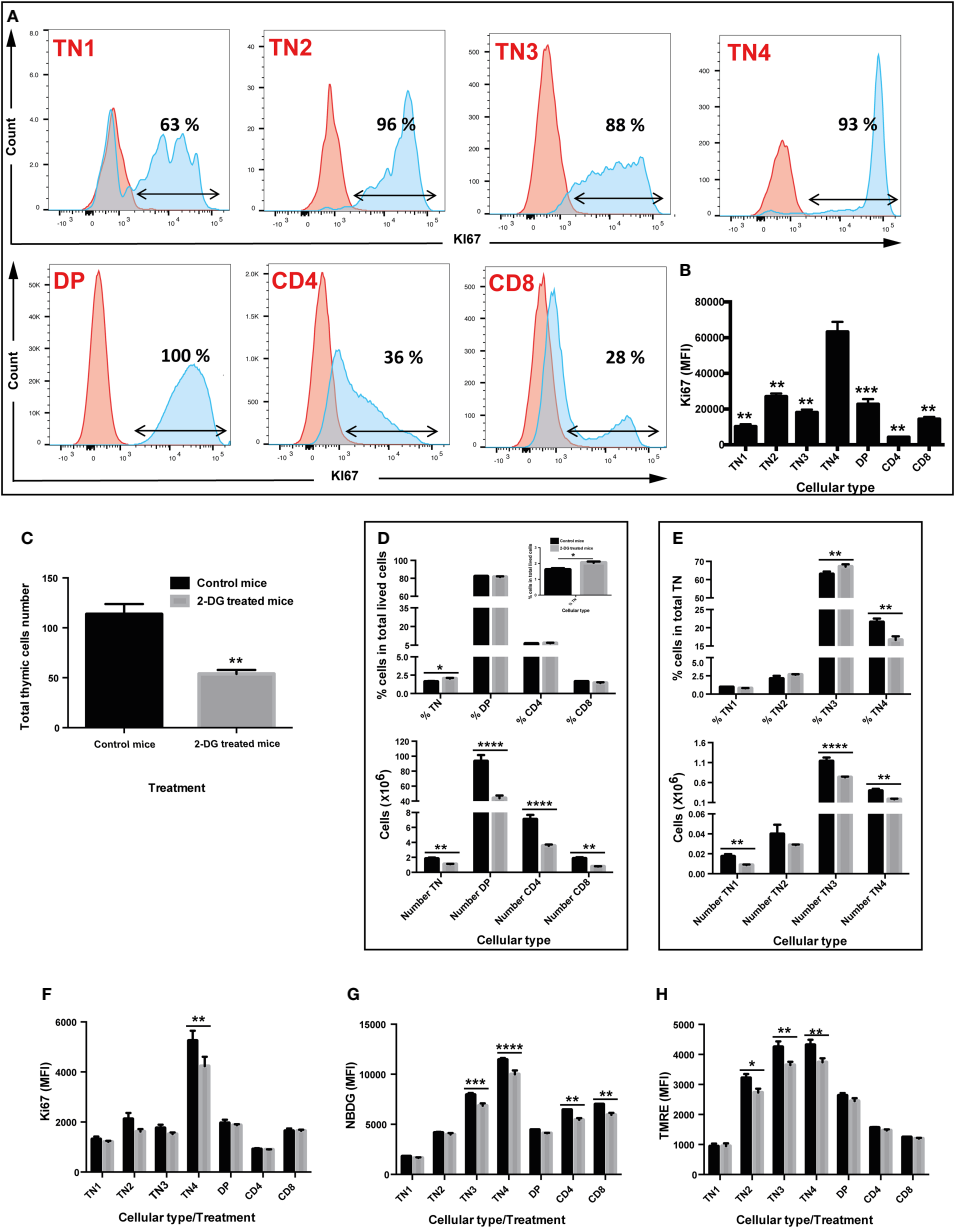
*in vivo* inhibition of glycolysis by 2-deoxyglucose (2-DG) interferes with proliferation, glucose absorption, and mitochondrial membrane potential of thymic progenitors, leading to a significant decrease in the total number of thymocytes without affecting their differentiation. (A & B) Cell proliferation of the different thymic lymphocyte populations. Cells (TN1, TN2, TN3, TN4, DP, SP CD4 and SP CD8) were labeled with an anti Ki67 antibody, a proliferation marker. **(A)** Flow cytometry histograms illustrating the percentage of cells that proliferate. The red curve corresponds to cells marked with the corresponding isotype of Ki67 and the blue curve to the cells labeled with the anti Ki67 antibody **(B)** Median fluorescence intensity of Ki67 labelled cells. **(C–H)** *in vivo* analysis of glycolysis inhibition by 2-DG. Two groups of 4 mice were used: group of control mice injected with PBS (black box) and mice injected with the 2-DG (grey box). **(C)** Total number of thymic cells. **(D)** Percentage (top graph) and number (bottom graph) of TN, DP, CD4 and CD8 cells in total living cells. **(E)** Percentage (top graph) and number (bottom graph) of TN1, TN2, TN3, TN4 cells in triple negative (TN) cells. **(F)** Quantification of proliferation by Ki67 median fluorescence intensity. **(G)** Quantification of glucose absorption by 2-NBDG median fluorescence intensity. **(H)** Analysis of mitochondrial membrane potential by TMRE median fluorescence intensity, marking active mitochondria. Data represent mean ± SEM (n=4 mice per group). Significance is indicated as follows: *p < 0.05, **p < 0.01, ***p < 0.001, ****p < 0.0001.

We showed that the TN3-TN4 pre-T cells exhibit the highest glycolytic function, with fused morphology and that very active mitochondria were present at theses stages. However, TN4 represented the highest proliferation level in the thymus (**Figures 5A, B**), which strongly correlates with the fact that TN4 cells displayed greater metabolic activity both in aerobic glycolysis and mitochondrial respiration.

To track the involvement of glycolysis in differentiation and/or thymic proliferation and given the importance of the glycolysis pathway in TN3 and TN4 cells, we inhibited this pathway in a more physiological context, *in vivo*. Two groups of 4 mice were treated by intraperitoneal injections of either PBS or 2-DG (2-deoxyglucose) at the dose of 750 mg/kg every 2 days for 2 weeks. *In vivo* treatment with 2-DG significantly reduced the number of total thymocytes by half (p<0.01) (control group: 113.5 million cells ±10.3 versus 53.8 million ±4 in the 2-DG-treated group) (**Figure 5C**). The proportions of DP thymocytes, CD4^+^TCRβ^+^ cells and CD8^+^TCRβ^+^ were not affected, whereas a small but significant increase in TN was observed (p<0.05) (**Figure 5D**, top panel). Among TN cells, the frequency of TN3 increased (p<0.01), whereas the percentage of TN4 decreased (p<0.01), suggesting a developmental arrest at this stage and/or reduced TN4 proliferation (**Figure 5E**, top panel). In addition, although the *in vivo* treatment with 2-DG did not have much effect on cellular viability, it significantly reduced: 1) cell proliferation, depicted by the MFI of Ki67, specifically in TN4 progenitors (p<0.01) (**Figure 5F**); 2) glucose absorption, measured by the MFI of NBDG, in TN3 cells (p<0.001), TN4 (p<0.0001), but also in CD4^+^TCRβ^+^ (p<0.01) and CD8^+^TCRβ^+^ (p<0.001) cells (**Figure 5G**); and 3) mitochondrial membrane potential, detected by the MFI of TMRE, specifically in TN2 (p<0.05), TN3 (p<0.01) and TN4 cells (p<0.01) (**Figure 5H**). In summary, the *in vivo* treatment of mice with 2-DG, reduced glycolysis as well as the membrane potential in TN3 and TN4 cells and resulted in a developmental arrest at the TN3 stage, thus affecting the TN3-TN4 transition. Moreover, proliferation was reduced in TN4 cells leading to a general reduction in thymus volume and decreased thymic cellularity.

### Defect in IL-7R signaling disturbs glucose absorption, mitochondrial membrane potential and proliferation in thymic T cell precursors

**Figure 6:**
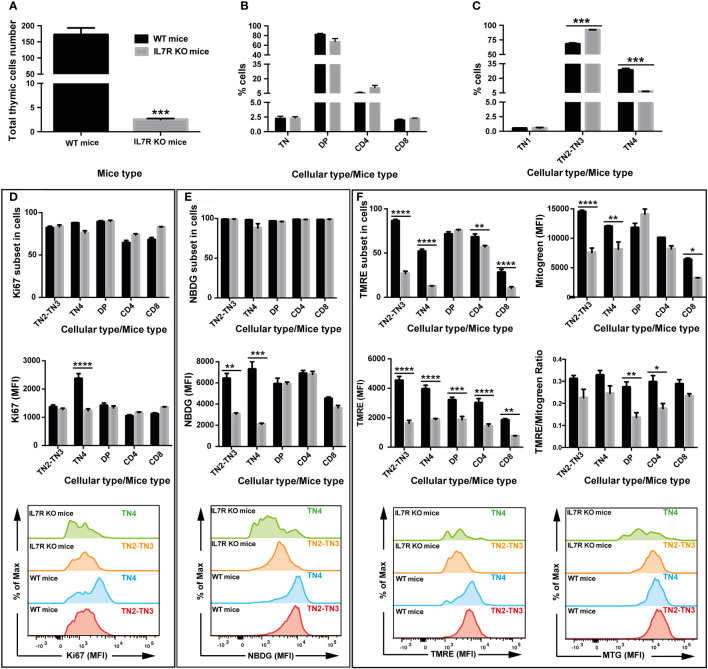
IL-7 signaling drives OXPHOS and glycolysis in T cell progenitors and correlates with the proliferation and the fate of T cells in the thymus: study in IL-7R deficient mice. Two groups of 4 mice were used: wild type mice (WT, black box) and group of Interleukin-7 receptor knockout mice (IL-7R KO, grey box). **(A)** Total number of thymic cells. **(B)** Percentage of TN, DP, CD4 and CD8 cells in total living cells. **(C)** Percentage of TN1, TN2, TN3, TN4 cells in TN cells. **(D)** Percentage of cells that express the proliferation marker Ki67 (top graph) and quantification of by Ki67 median fluorescence intensity (middle & bottom graphs). **(E)** Percentage of cells that absorb 2-NBDG (top graph) and quantification of glucose uptake by 2-NBDG median fluorescence intensity (middle & bottom graphs). **(F)** Percentage of cells that express TMRE, marking active mitochondria (top-left graph) and analysis of the mitochondrial membrane potential by TMRE median fluorescence intensity (middle- & bottom-left graphs). Quantification of mitochondrial mass by Mitotracker Green median fluorescence intensity (top- & bottom-right graphs). Analysis of the mitochondrial membrane potential/mitochondrial mass ratio expressed as TMRE/Mitotracker Green ratio (middle-right graph). Data represent mean ± SEM of 3 to 4 mice per group from 3 independent experiments. Significance is indicated as follows: *p < 0.05, **p < 0.01, ***p < 0.001, ****p < 0.0001.

Based on our findings on TN3 and TN4 pre-T cells where glucose inhibition led to reduced thymic growth, we used a mouse model whose thymic expansion was altered due to the absence of IL-7 signaling. IL-7 is a powerful agent supporting survival and proliferation, and its receptor is expressed by lymphocytes (45). Mice deficient in IL-7 or IL-7 receptor (IL-7R) have reduced thymic cells number and altered T cell development (46–48). In our IL-7R deficient mice, the total thymocyte number was reduced by about 42 times compared to controls (control group: 208 million cells ± 17 versus 4.9 million ± 1.8 in the IL-7R-deficient group) (**Figure 6A**). Frequencies of DP thymocytes, CD4^+^TCRβ^+^ cells and CD8^+^TCRβ^+^ were not affected in the wild-type (WT) group or the IL-7R deficient group (**Figure 6B**). Analysis of TN thymocytes with CD44 and CD25 markers revealed that the frequency of TN2-TN3 cells was increased in IL-7R deficient mice, whereas the percentage of TN4 cells decreased (**Figure 6C**), resulting in a significant blockage at the TN3 stage, thus affecting the TN3-TN4 transition. Moreover, these TN4 progenitors had reduced proliferation, as revealed by the Ki67 MFI measurements (**Figure 6D**, top and bottom panel respectively). However, an IL-7-independent pathway exists leading to the generation of DP and SP thymocytes (12). In addition, the total number of all progenitor subsets, DP, CD4 and CD8 was reduced.

While glucose absorption was similar in all subsets in IL-7R KO thymi (**Figure 6E**, top panel), the MFI of NBDG, showed a significant reduction specifically in TN2-TN3 and TN4 pre-T cells (**Figure 6E**, bottom panel). Thus, TN4 pre-T IL-7R KO cells are highly dependent on glucose for their proliferation (absorption and proliferation were decreased); in contrast, IL-7R KO TN2-TN3 cells presented defective glucose absorption, but proliferated within the same range as TN2-TN3 control cells, by a glucose-independent process (glucose affected, proliferation unchanged). In addition, analysis of mitochondrial membrane potential showed a marked decrease in the percentage of cells uptaking TMRE in the IL-7R deficient group compared to the control group in all thymic lymphocytic populations, except DP cells (**Figure 6F**, top panel), with a significant MFI reduction in all thymic lymphocytic populations (**Figure 6F**, bottom panel). Thus, Il-7 signaling supports the proliferation in TN4 pre-T cells, probably by driving glucose absorption and mitochondrial membrane potential.

## Discussion

The maturation of thymocytes faces major metabolic challenges and highly regulated mitochondrial dynamics, that are closely linked to each other, at the time of differentiation/proliferation. We show for the first time that TN3-TN4 progenitors display very active mitochondria, with a fused morphology and rely on high glycolysis and OXPHOS. Following their differentiation towards mature thymic T cells (DP and SP), mitochondria become fragmented and metabolically less active, as revealed by the expression of several metabolites, using a targeted metabolomic approach. Mature cells show lower basal ECAR and OCR compared to TN, especially in DP where the lowest values were observed. Our study presents for the first time, metabolomic data during thymic cell differentiation and maturation, and our results are in line with those described recently by V. Sun et al. (49) showing that the metabolic profiles (using a transcriptomic approach), specifically OXPHOS and glycolysis, undergo dramatic metabolomic changes between TN, DP and SP stages in murine and human thymi.

The hyperfused mitochondria of TN could be explained by the fact that these cells are primarily activated cells that undergo metabolic reprogramming to provide energy and important molecular building blocks for growth, proliferation, and differentiation, using a coordinated action of aerobic glycolysis and mitochondrial oxidative phosphorylation.

Our observations are in keeping with a recent paper by Yao Cong-Hui (50), which compares cellular metabolism in the same cells in quiescent and proliferative state. The authors found that although fibroblast proliferation had improved glycolysis, these cells also increased oxidative phosphorylation two-fold and increased their mitochondrial coupling efficiency by 30%. Interestingly, the authors found that both increases were supported by mitochondrial fusion, as we found in our TN1, TN2, TN3 and TN4 cells. Moreover, our results partially recapitulate those from two studies (51, 52) showing that when hematopoietic stem cells (HSCs) are in active division, like in TN cells, their mitochondria are hyperfused and rely on glycolysis, as opposed to HSCs with a quiescent state, which had small, highly motile and fragmented mitochondria and a downregulation of glycolysis, maintaining a low metabolic activity.

Accordingly, our *in vitro* inhibition of mitochondrial fission during progenitor differentiation, affected the TN to DP transition, leading to a decrease in the total number of thymic cells. Our results recall those obtained by Corrado et al. and Simula et al., showing that either a deletion in mitochondrial fission protein Drp1 (4) or mitochondrial fusion protein Opa1 (23), led to an imbalance between these two processes, thus leading to a reduction in the number of thymocytes, probably by affecting their proliferation/differentiation and their metabolic reprogramming.

By using the LC-MS based analysis of TN3 and TN4 metabolome for the first time, we showed that these subsets are distinct: TN3 rely more on oxidative phosphorylation whereas TN4 are more glycolytic. In line with this, TN4 strongly express HK2 in comparison to TN3, supporting the fact that TN4 cells are more glycolytic than TN3. We showed that TN4 proliferate much more than TN3 and other differentiated thymocytes, and this proliferation correlates with the fact that TN4 display greater glycolysis and mitochondrial respiration. Concordantly, treatment of mice with 2-DG reduced glycolysis as well the mitochondrial membrane potential in TN3 and TN4 cells, affecting the TN3-TN4 transition. The proliferation was reduced in TN4, and led to a general decrease by half in total number of cells. The reduction in mitochondrial membrane potential relates also to the mitochondrial ability to generate ATP by oxidative phosphorylation in TN4 cells. Indeed, glycolysis describes a series of enzymatic reactions that generate pyruvate from glucose. The major metabolic fate of pyruvate is its importation into the mitochondria and its conversion into acetyl-CoA which enters the TCA cycle to produce NADH and FADH_2_ which can later be used to generate ATP through OXPHOS (53, 54). Moreover, glycolysis and TCA cycle should be seen as a metabolic route for anabolic biosynthetic reactions that play an important role in proliferation, suggesting that 2-DG may inhibit proliferation via restricting cellular and mitochondrial metabolism in TN4 cells. Our results are in line with those observed in the N-diethylnitrosamine (DEN)-induced rat hepatocarcinoma model treated with 2-DG, showing a decrease in: cell proliferation, glycolysis products, TCA cycle, fatty acid and cholesterol biosynthesis, and ATP production, leading to delayed hepatocarcinogenesis, suggesting that 2-DG may inhibit hepatocarcinogenesis in DEN-treated rats via restricting cancer cell metabolism (55).

Based on our results that glycolysis inhibition leads to reduced thymi, we used the IL-7R KO mice, whose thymic expansion is greatly altered. Our results reveal for the first time a reduction in glycolysis as well in the mitochondrial membrane potential in TN3 and TN4 cells, affecting the TN3-TN4 transition. TN4 proliferation was reduced, leading to a 40-fold general decrease in total number of cells. This is consistent with a glucose/IL-7R-dependent proliferation of TN4 pre-T cells. Several essential metabolic targets of the IL-7 signal transduction pathway have been characterized *in vitro*. Glucose metabolism is a multi-step process that could be regulated by IL-7 at different levels. A mechanism by which IL-7 could control glucose metabolism in T-cells is through increased traffic of glucose transporter Glut1, the main glucose transporter in T cells (56), to the cell surface, since IL-7 has been shown to promote Glut1 traffic via STAT-5-mediated Akt activation to support cell survival (57).

The activity of other factors involved in glycolysis could be regulated by IL-7. Possible targets are HKs or PFK-1 that control two key stages of glycolysis. Indeed, it has been shown that IL-7 deprivation *in vitro*, causes a rapid decline in glucose metabolism that correlates with a decrease in HK2 expression, using cell lines dependent on IL-7 and primary lymphocytes (40). The re-addition of IL-7 to deprived lymphocytes restored HK2 at a transcriptional and protein level. Inhibition of HK2 activity by chemical inhibitors or RNA interference decreases glucose use as well as ATP levels, in the presence of IL-7; however, its overexpression restores glucose retention.

We demonstrate that the decline in glucose metabolism caused by IL-7 deprivation led to a decrease in membrane potential in all thymic lymphocyte populations. Indeed, the reduction in glycolysis products could affect the TCA cycle and ATP production contributing to a decrease in mitochondrial membrane potential which underscores a decrease in mitochondrial ability to generate ATP. This decrease in membrane potential could also be due to a decrease in the β-oxidation of fatty acids, which serve as electron donors in mitochondria to generate ATP, and thus a decrease in the production of ATP. Indeed, it has been demonstrated that IL-7R-mediated signaling induces fatty acid oxidation-dependent oxidative phosphorylation in naive CD8^+^ T cells (58). Moreover, it is possible that the activation of IL-7R results in the phosphorylation of STAT5 (pSTAT5) (59), and its subsequent translocation to the nucleus, leading to transcriptional activation of glycolytic and fatty acid metabolism genes (60, 61). In the absence of IL-7, the decrease in the cell proliferation could be attributed to a reduction of fatty acid oxidation which is known to impair *de novo* nucleotide synthesis for DNA replication (62).

Our results show that IL-7 signaling, during TN3 and TN4 stages, promote proliferation probably by driving glucose absorption and the mitochondrial membrane potential. Our results complete and concord with those observed by Boudil et al. (12) showing that IL-7 signaling, during TN3 and TN4 stages, activates PI(3)K and expression of the gene encoding the PI(3)K isoform required for β-selection; it also promotes expression of nutrient-transport proteins such as CD98 (an AA transporter) and CD71 (a transferrin receptor). All these combined activities promote the growth and proliferation of cells. In addition, IL-7 signaling inhibits the transcriptional repressor Bcl-6 and thereby repress the differentiation of TN3 and TN4 thymocytes and allows greater expansion of theses populations.

Our study allows us to establish a mitochondrial mapping of each thymic cell populations, by establishing a correlation between mitochondrial morphology, energy metabolism, proliferation and differentiation of these cells, which is controlled by IL-7 and Notch signaling. Indeed, Notch signaling pathway has an essential role in the development of early stages of thymic T cells at β-selection checkpoint (10, 31, 63–65). It plays a key role in the regulation of cellular metabolism (9). Moreover, together with IL-7, PI3K and mTORC signaling, Notch determines proliferation and T cell lineage commitment (9, 12, 16). In addition, mitochondrial fusion directs cardiomyocyte differentiation via calcineurin and Notch signaling (66), suggesting that, this connection between mitochondrial form and Notch signaling may occur in thymocyte development.

In conclusion, our findings suggest that TN cells differ from DP and SP cells by their metabolism and mitochondrial morphology which correlate with their proliferation and/or differentiation status. Moreover, modifications in the mitochondrial metabolism or morphology at progenitor stages led to a direct decrease in cell number, proliferation and/or differentiation. Hence, our analysis shed light on two metabolic statuses within thymic subsets associated with two distinct morphologies that could be targeted in order to improve T cell-based therapies.

## Data availability statement

The metabolome datasets presented in this study can be found in online repositories. The names of the repository/repositories and accession number(s) can be found below: MTBLS8340 (Metabolights).

## Ethics statement

The animal studies were approved by Paris Descartes University Ethical Committee and the French Ministry of Research, Innovation and Education under the following reference APAFIS #26599-2019071812345604. The studies were conducted in accordance with the local legislation and institutional requirements. Written informed consent was obtained from the owners for the participation of their animals in this study.

## Author contributions

RE: Conceptualization, Formal analysis, Funding acquisition, Investigation, Project administration, Resources, Supervision, Validation, Visualization, Writing – original draft, Writing – review & editing. MK: Investigation, Visualization, Writing – review & editing. NG: Formal analysis, Investigation, Resources. JM: Formal analysis, Investigation, Resources. AL: Investigation. IN: Formal analysis, Investigation, Resources. CP: Investigation, Resources. VQ: Investigation. TW: Conceptualization, Resources, Writing – review & editing. AH: Conceptualization, Formal analysis, Funding acquisition, Investigation, Project administration, Resources, Supervision, Validation, Visualization, Writing – original draft, Writing – review & editing, Project administration, Resources, Visualization, Funding acquisition. SE: Conceptualization, Funding acquisition, Project administration, Conceptualization, Writing – review & editing, Writing – original draft, Writing – review & editing.

## Supplementary material

Supplementary Figure 1 Strategy for analyzing and sorting of thymic T cell populations. Thymocytes are gated as CD4^-^CD8^-^ DN, CD4^+^CD8^+^ DP, CD4^+^TCRβ^+^ SP, CD8^+^TCRβ^+^ SP and CD8^+^TCRβ^-^ ISP (CD8 immature -single positive cells). Among DN, by staining the cells with LIN, a cocktail of antibodies containing CD11b, CD11c, NK1.1, Ly6G/Ly6C, CD19, TCRβ, TCRγδ and Ter119, we gated the LIN^-^ CD25^+^ DN cells. Among these cells, TN cells were characterized using CD44 and CD25 antibodies: TN1 (CD44^+^CD25^-^), TN2 (CD44^+^CD25^+^), TN3 (CD44^-^CD25^+^) and TN4 (CD44^-^CD25^-^).

Supplementary Figure 2 3D image reconstruction showing examples of each mitochondrial morphology in cells sorted from Mito-YFP mice. Left panel represents a cell with fused mitochondria (small and round shape, pink arrows), right panel a cell with fragmented mitochondria (elongated shape and network, yellow arrows) and middle panel a cell with both morphologies (pink and yellow arrows).

Supplementary Figure 3 Modulation of mitochondrial dynamics of TN3 cells cultured in the presence of mitochondrial fusion promoter M1 affected the TN-DP transition and decreased the total number of differentiated thymocytes. TN3 cells co-cultured *in vitro* on OP9-DL4 feeder cells, in the presence of M1 (10 μM and 20 μM). M1 was added at day 0 (D0) and every 3 days for 10 days. Cells were analyzed on day 6 (D6) and day 10 (D10). For each, the cells were marked with a cocktail of antibodies (anti-CD4, anti-CD8, anti-TCRβ, anti-LIN, anti-CD44 and anti-CD25) to assess the percentage of living cells at D6 **(A)** and D10 **(C)**, TN, DP, SP CD4^+^ TCRβ^+^ and SP CD8+ TCRβ^+^ at D6 **(E)** and D10 **(G)**, TN3 and TN4 at D6 **(I)** and D10 **(K)** and the absolute number of the living cells at D6 **(B)** and D10 **(D)**, TN, DP, SP CD4^+^ TCRβ^+^ and SP CD8^+^ TCRβ^+^ at D6 **(F)** and D10 **(H)**, TN3 and TN4 at D6 **(J)** and D10 **(L)**. Data from a representative experiment (n=3 independent experiments). Each experiment represents average ± SEM of 3 individual wells. Statistical significance is indicated as follows: *p < 0.05, **p < 0.01, ***p < 0.001, ****p < 0.0001.

Supplementary Movie 1 3D video reconstruction showing examples of each mitochondrial morphology in cells sorted from Mito-YFP mice. Left panel represents a cell with fused mitochondria (small and round shape), right panel a cell with fragmented mitochondria (elongated shape and network) and middle panel a cell with both morphologies.

Supplementary Table 1 Differentially expressed metabolites between the different thymocyte populations. One-way analysis of variance for individual metabolites in different cell populations (ANOVA test with Fischer’s LSD *post hoc* test). 115 metabolites were retained for analysis and 87 are differentially expressed (statistically significant: p < 0.05), highlighted in blue. Data are representative of 5 to 6 samples for each cell population, from 2 independent experiments.
