# Supplementary figures and images for: Mitochondrial dynamics and metabolic regulation control T cell fate in the thymus
Source: Front Immunol. 2024 Jan 15;14:1270268. doi: 10.3389/fimmu.2023.1270268 (PMC10822881; doi:10.3389/fimmu.2023.1270268)

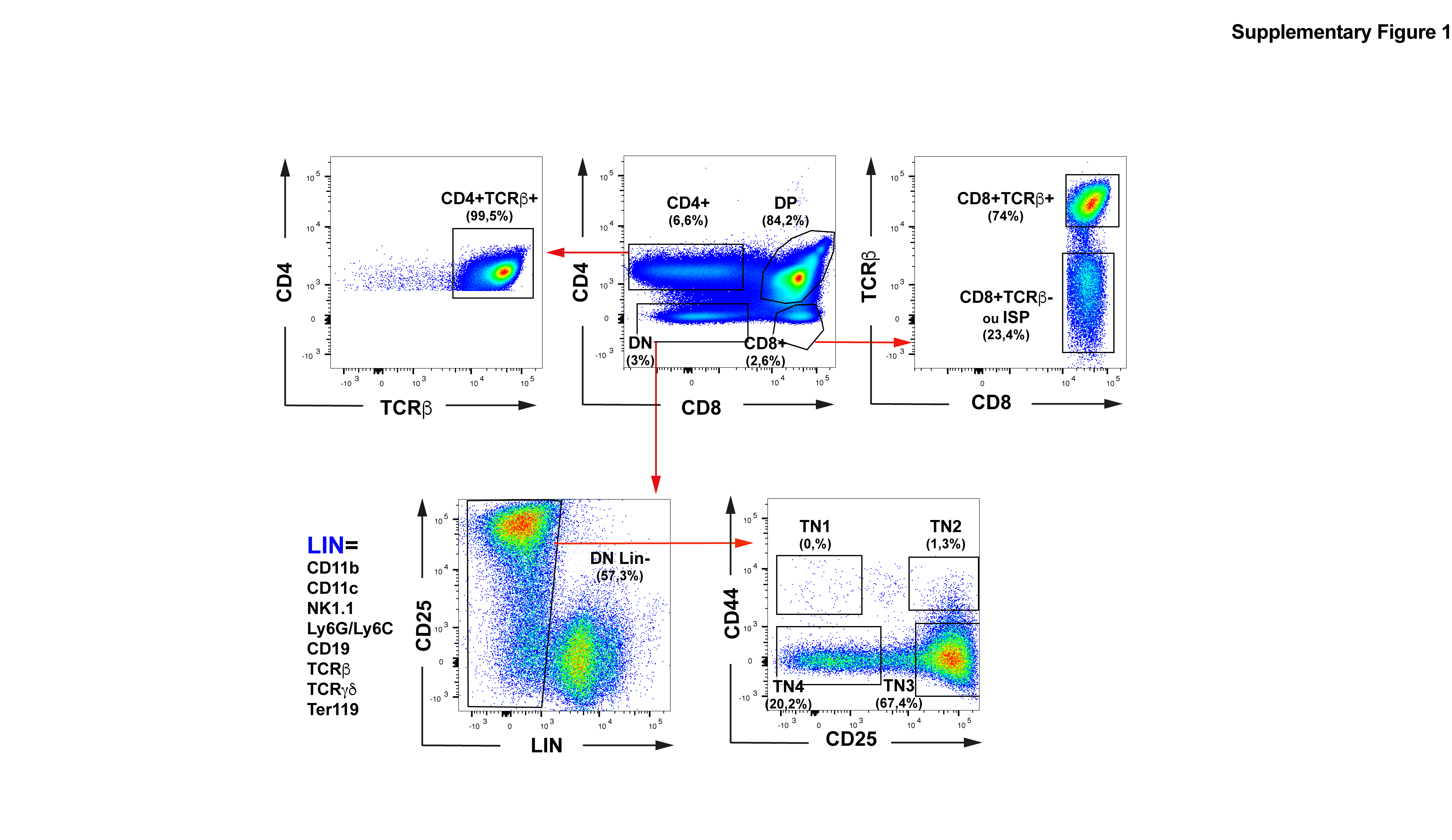

Supplement: Supplementary Figure 1 — Strategy for analyzing and sorting of thymic T cell populations. Thymocytes are gated as CD4-CD8- DN, CD4+CD8+ DP, CD4+TCRβ+ SP, CD8+TCRβ+ SP and CD8+TCRβ- ISP (CD8 immature -single positive cells). Among DN, by staining the cells with LIN, a cocktail of antibodies containing CD11b, CD11c, NK1.1, Ly6G/Ly6C, CD19, TCRβ, TCRγδ and Ter119, we gated the LIN- CD25+ DN cells. Among these cells, TN cells were characterized using CD44 and CD25 antibodies: TN1 (CD44+CD25-), TN2 (CD44+CD25+), TN3 (CD44-CD25+) and TN4 (CD44-CD25-). [file Image_1.tif]

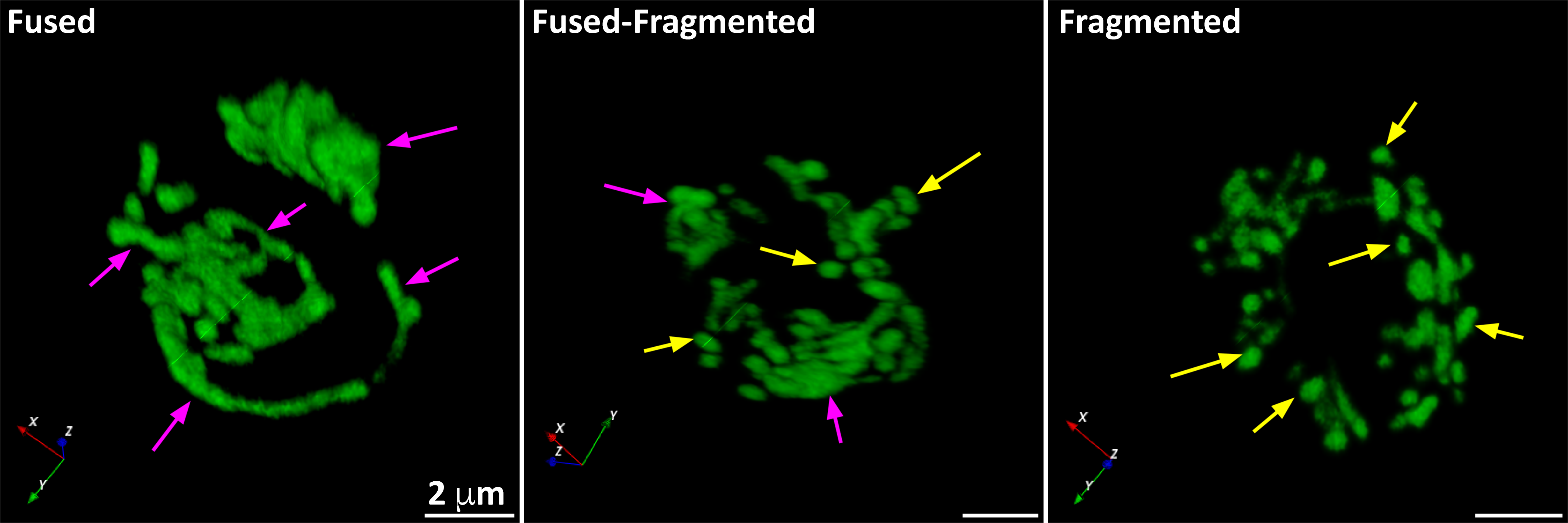

Supplement: Supplementary Figure 2 — 3D image reconstruction showing examples of each mitochondrial morphology in cells sorted from Mito-YFP mice. Left panel represents a cell with fused mitochondria (small and round shape, pink arrows), right panel a cell with fragmented mitochondria (elongated shape and network, yellow arrows) and middle panel a cell with both morphologies (pink and yellow arrows). [file Image_2.tif]

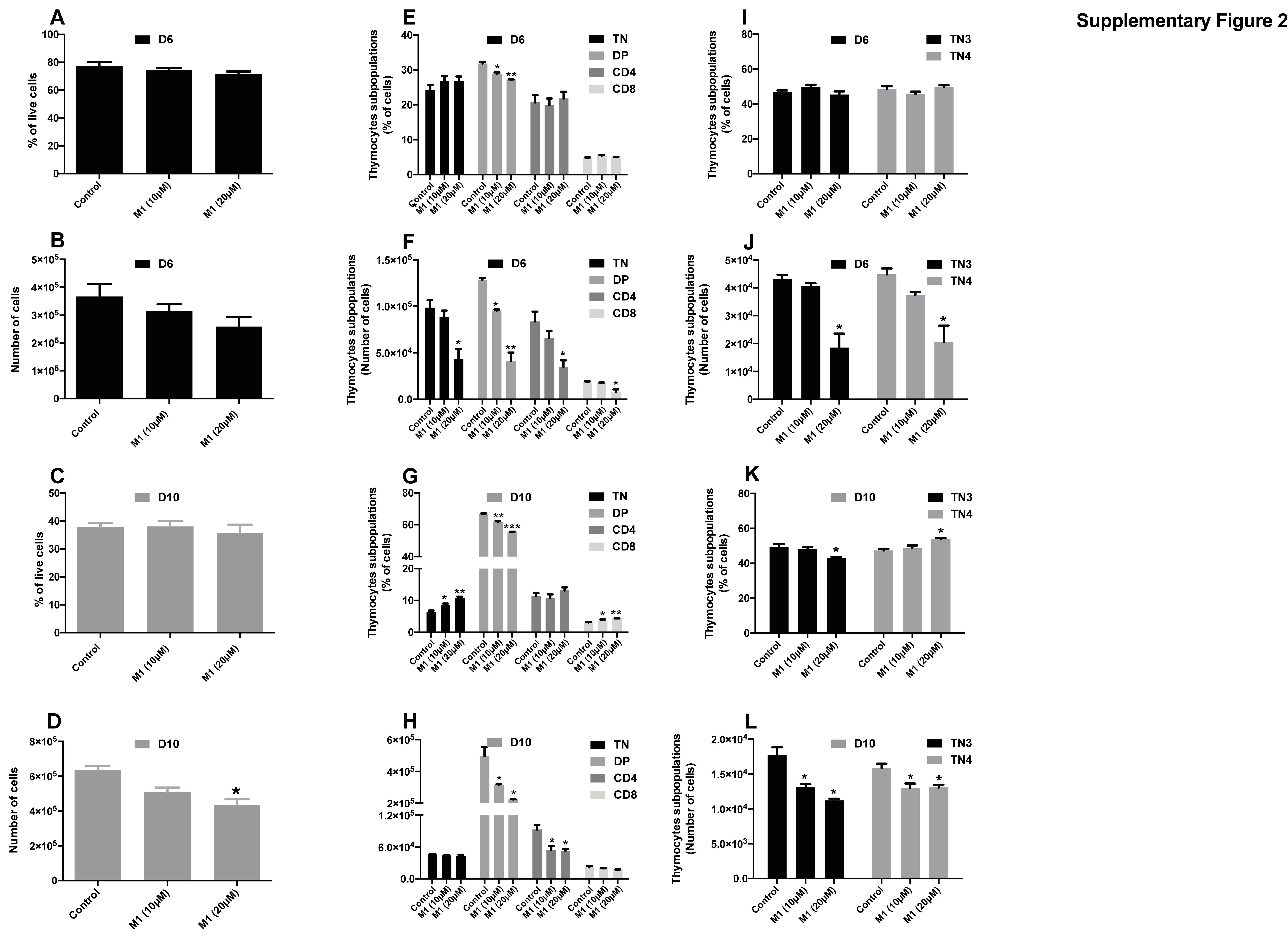

Supplement: Supplementary Figure 3 — Modulation of mitochondrial dynamics of TN3 cells cultured in the presence of mitochondrial fusion promoter M1 affected the TN-DP transition and decreased the total number of differentiated thymocytes. TN3 cells co-cultured in vitro on OP9-DL4 feeder cells, in the presence of M1 (10 μM and 20 μM). M1 was added at day 0 (D0) and every 3 days for 10 days. Cells were analyzed on day 6 (D6) and day 10 (D10). For each, the cells were marked with a cocktail of antibodies (anti-CD4, anti-CD8, anti-TCRβ, anti-LIN, anti-CD44 and anti-CD25) to assess the percentage of living cells at D6 (A) and D10 (C), TN, DP, SP CD4+ TCRβ+ and SP CD8+ TCRβ+ at D6 (E) and D10 (G), TN3 and TN4 at D6 (I) and D10 (K) and the absolute number of the living cells at D6 (B) and D10 (D), TN, DP, SP CD4+ TCRβ+ and SP CD8+ TCRβ+ at D6 (F) and D10 (H), TN3 and TN4 at D6 (J) and D10 (L). Data from a representative experiment (n=3 independent experiments). Each experiment represents average ± SEM of 3 individual wells. Statistical significance is indicated as follows: *p < 0.05, **p < 0.01, ***p < 0.001, ****p < 0.0001. [file Image_3.tif]
